# Staurosporine induces apoptosis in pancreatic carcinoma cells PaTu 8988t and Panc-1 via the intrinsic signaling pathway

**DOI:** 10.1186/s40001-019-0365-x

**Published:** 2019-01-28

**Authors:** Manuela Malsy, Diane Bitzinger, Bernhard Graf, Anika Bundscherer

**Affiliations:** Department of Anesthesiology, University Medical Center Regensburg, Franz Josef Strauss Allee 11, 93053 Regensburg, Germany

**Keywords:** Staurosporine, Apoptosis, Pancreatic carcinoma, Colorectal carcinoma, Cancer

## Abstract

**Background:**

Cancer, one of the leading causes of death worldwide, develops when the normal balance between mitosis and apoptosis is disrupted. The subsequently increased proliferation rate or decreased apoptosis rate of cells leads to uncontrolled cellular growth. Thus, the current aim of cancer research is to increase the apoptosis rate in tumor cells—while limiting the concurrent death of healthy cells—and to induce controlled apoptosis in abnormal cells. Staurosporine is a very potent inducer of apoptosis because it inhibits many different kinases. So far, many different kinase pathways of staurosporine-induced apoptosis have been discussed for various tumor entities.

**Aims:**

To identify the effect of staurosporine in pancreatic and colorectal carcinoma cells and its apoptosis-inducing signaling pathway.

**Methods:**

The apoptosis rate in pancreatic and colorectal carcinoma cells was analyzed by annexin V staining after staurosporine administration. Staurosporine stimulation and its effects on the expression of Bcl2, BAX, Bad, caspase-8, and caspase-9 were investigated with immunoblot.

**Results:**

Staurosporine significantly increased apoptosis in pancreatic carcinoma cells. Western blot analysis showed activation of caspase-9 in PaTu 8988t and Panc-1 cells with 1 µM staurosporine. In addition, expression of Bcl2 and Bad was decreased in PaTu 8988t cells. In colorectal carcinoma cells SW 480, staurosporine stimulation did not induce apoptosis.

**Conclusion:**

Modern therapeutic strategies for tumor diseases target the efficient modulation of specific signaling and transcription pathways. In this respect, the therapeutic potential of protein kinase inhibitors has been repeatedly discussed. Our study showed that staurosporine induces apoptosis in pancreatic carcinoma cells via the intrinsic signaling pathway. Thus, staurosporine is a suitable positive control for in vitro apoptosis tests for the pancreatic cancer cell lines PaTu 8988t and Panc-1. Further clinical studies should analyze the impact of this finding on cancer treatment.

## Background

Malignant tumors are one of the main scourges of humanity. In 2012 alone, about 8.2 million people died of carcinoma worldwide, and—according to the World Health Organization—[1] this figure is expected to rise to 13 million over the next 20 years.

From the molecular biology point of view, cancer is defined as any type of malignant neoplasm, independent of the organ or the tissue from which the tumor originates [2]. Cancer develops when the normal balance between mitosis and apoptosis is disrupted [3]. The subsequent increase in the proliferation rate or the decrease in the apoptosis rate of cells results in uncontrolled cellular growth [4, 5]. Thus, the current aim of cancer research is to increase the apoptosis rate in tumor cells—while simultaneously limiting concurrent death of healthy cells—and to induce controlled apoptosis in abnormal cells [6].

**Fig. 1:**
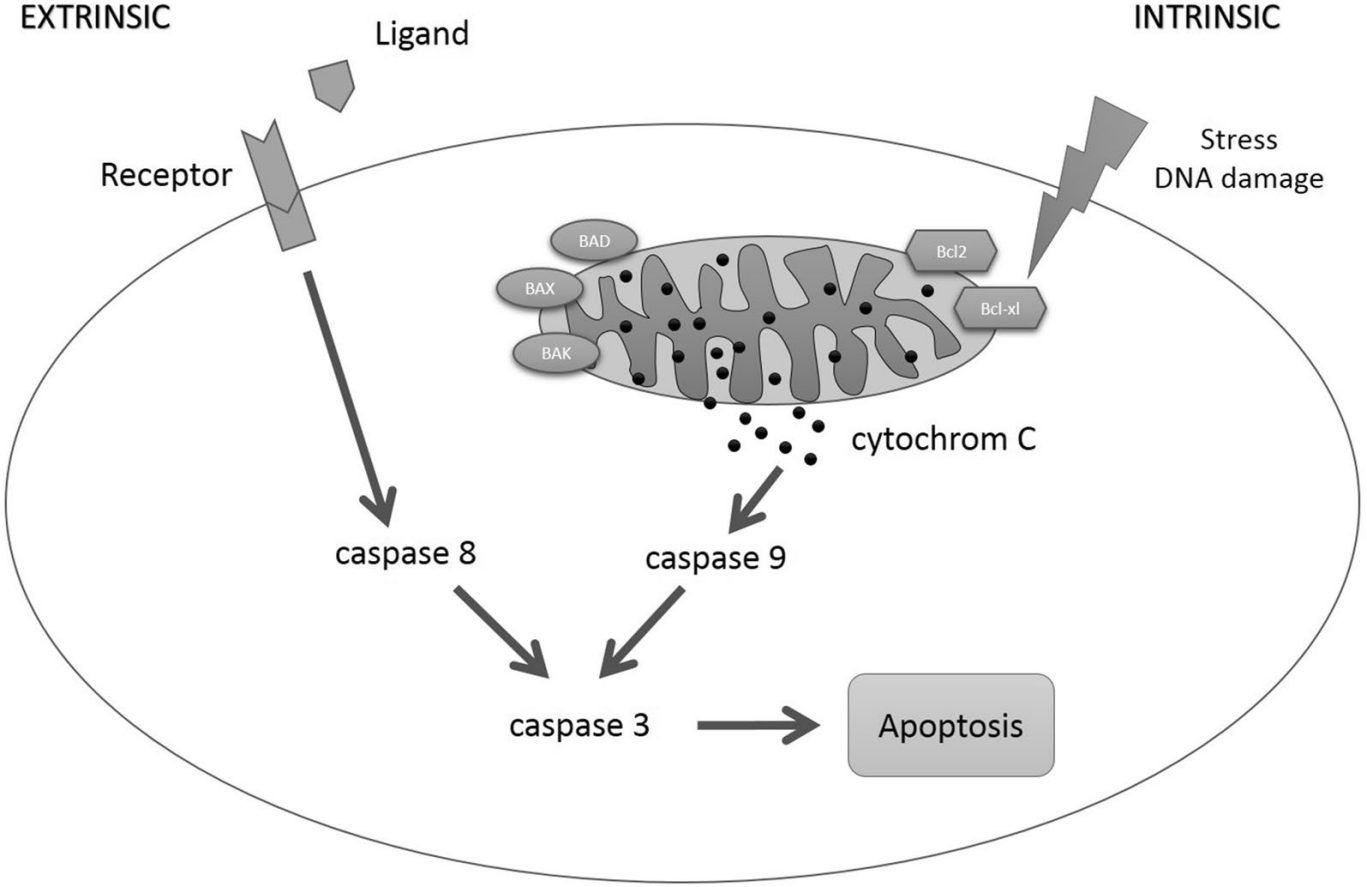
Apoptotic signaling pathway. Caspase-8 is activated via the extrinsic signaling pathway by the binding of a ligand to the death receptor. The intrinsic signaling pathway is activated by changes in the mitochondrial membrane potential and the subsequent release of cytochrome C, which influences the pro-apoptotic factors BAK, Bad, and BAX as well as the anti-apoptotic factors Bcl2 and Bcl-xl and thus triggers caspase-9. Both pathways merge into a common pathway, in which effector caspase-3 finally induces apoptosis

Therefore, comprehensive knowledge of the mechanisms of apoptosis in the different types of cells is vital for developing potential cancer therapies [7]. The term apoptosis refers to programmed cell death, which constitutes an important mechanism for maintaining tissue homeostasis [8]. The characteristic feature in this respect is that apoptosis is actively induced by the respective cell itself; thus, the cell is part of the metabolism. Activation of proteolytic enzymes termed caspases results in cellular shrinkage, condensation, and fragmentation of the cell nucleus, loss of cellular adhesion, and finally in apoptosis [9]. Apoptosis is induced by activation of important signal-transduction cascades [10]. Here, extrinsic and intrinsic signaling pathways have to be differentiated [11]. After the binding of a ligand to the death receptor of the tumor necrosis factor receptor family CD 95 (APO-1/Fas) or TRAIL receptors, caspase-8 is activated via the extrinsic signaling pathway [12, 13]. In turn, caspase-8 activates caspase-3 (effector caspase) [14], thus inducing apoptosis. The intrinsic signaling pathway is the cellular response to stress [15]. Changes in the mitochondrial membrane result in the collapse of its potential and thus in the release of cytochrome C, which subsequently irreversibly instigates the entire caspase cascade by activating caspase-9 [16] (Fig. 1).

A highly potent inductor of apoptosis is staurosporine, an alkaloid that inhibits many different kinases [17]. Several new derivatives of this substance, which was originally isolated from *Streptomyces staurosporeus*, have been synthesized in clinical trials to be used in anticancer therapies [18]. So far, many different kinase pathways of staurosporine-induced apoptosis have been discussed for various tumor entities [19–21].

The aim of this study was to identify the effect of staurosporine in pancreatic and colorectal carcinoma cells and its apoptosis-inducing signaling pathway.

## Methods

### Cell lines

The human pancreatic adenocarcinoma cell lines PaTu 8988t and Panc-1 were obtained from Professor Ellenrieder (Philipps University of Marburg, Germany). The colorectal carcinoma cell line SW 480 was purchased from the German Collection of Microorganism and Cell Culture (DSMZ). PaTu 8988t and Panc-1 cells were maintained in Dulbecco’s modified Eagle’s medium (Sigma-Aldrich, Steinsheim, Germany) or RPMI 1640 (Pan Biotech, Aidenbach, Germany), which was supplemented with 10% fetal calf serum (FCS) (Sigma-Aldrich) and 5% Myco Zap (Lonza Verviers SPRL, Verviers, Belgium) or 5% penicillin plus streptomycin (Sigma-Aldrich) for SW 480. Cells were cultured at 37 °C in humidified CO_2_ atmosphere (5%) and maintained in monolayer culture. Experiments were done with cells at ~ 70–80% confluence.

### Antibodies and reagents

Staurosporine was purchased from Sigma-Aldrich. Final concentrations were achieved by diluting drugs in standard growth media. All solutions were prepared freshly prior to use. For immunoblotting, membranes were probed with antibodies against Bcl2, BAX, Bad, caspase-8, caspase-9 (cell signaling), and ß-actin (Sigma-Aldrich).

### Subcellular fractionation and immunoblotting

Cells were washed twice with cold DPBS and collected by centrifugation at 4000 rpm at 4 °C for 10 min. Lysates were then resuspended in RIPAE-buffer (5 mL Triton X-100, 190 mg EDTA, 0.5 g SDS, 2.5 g deoxycholic acid, 500 mL DPBS, protease inhibitors) for 15 min and centrifuged at 13,000 rpm for 30 min. The supernatants were transferred to new cups and incubated on ice. 30 µg of the total lysates were analyzed by SDS-PAGE and blotted onto nitrocellulose. After protein extraction and gel transfer, the membranes were washed in TBS washing buffer and incubated with peroxidase-conjugated secondary antibodies. Immunoreactive proteins were visualized by means of an enhanced chemiluminescence detection system (Western Blotting Detection Reagent, GE Healthcare).

### Apoptosis analysis

Apoptosis assays by annexin V staining were conducted according to the manufacturer’s instructions (BD Pharming). In brief, PaTu 8988t, Panc-1, and SW 480 cells were incubated with 1 µM of staurosporine. Standard growth medium was used for negative control. After 0 h, 3 h, 6 h, 9 h, 12 h, 16 h, or 24 h incubation time, the supernatant was decanted from the cells to preserve floating cells. Adherent cells were rinsed with warm PBS (Sigma-Aldrich) and harvested by standard trypsinization. Afterward, harvested and floating cells were mixed, washed, and resuspended in binding buffer at a final concentration of 106 cells/ml. 100 µL of cell suspension containing 105 cells was resuspended in 5 µL of FITC Annexin plus 5 µL of propidium iodide, followed by 15 min incubation at room temperature protected from light. The cells were washed and resuspended with 400 µL of binding buffer. Finally, the cells were analyzed by flow cytometry using FACS Calibur (BD Bioscience) and CellQuest Pro software (BD Bioscience). All tests were done in duplicate and the process was repeated twice.

### Statistical analysis

Data are presented as mean ± SD. The non-parametric Mann–Whitney *U* test was used for statistical evaluation of the data. *P* values < 0.05 were considered significant. IBM SPSS Statistics (Vs. 20; IBM New York, US) and Excel Vs. 2010 (Microsoft, Redmond, USA) packages were employed for statistical analysis.

## Results

### Analysis of apoptosis and necrosis

**Fig. 2:**
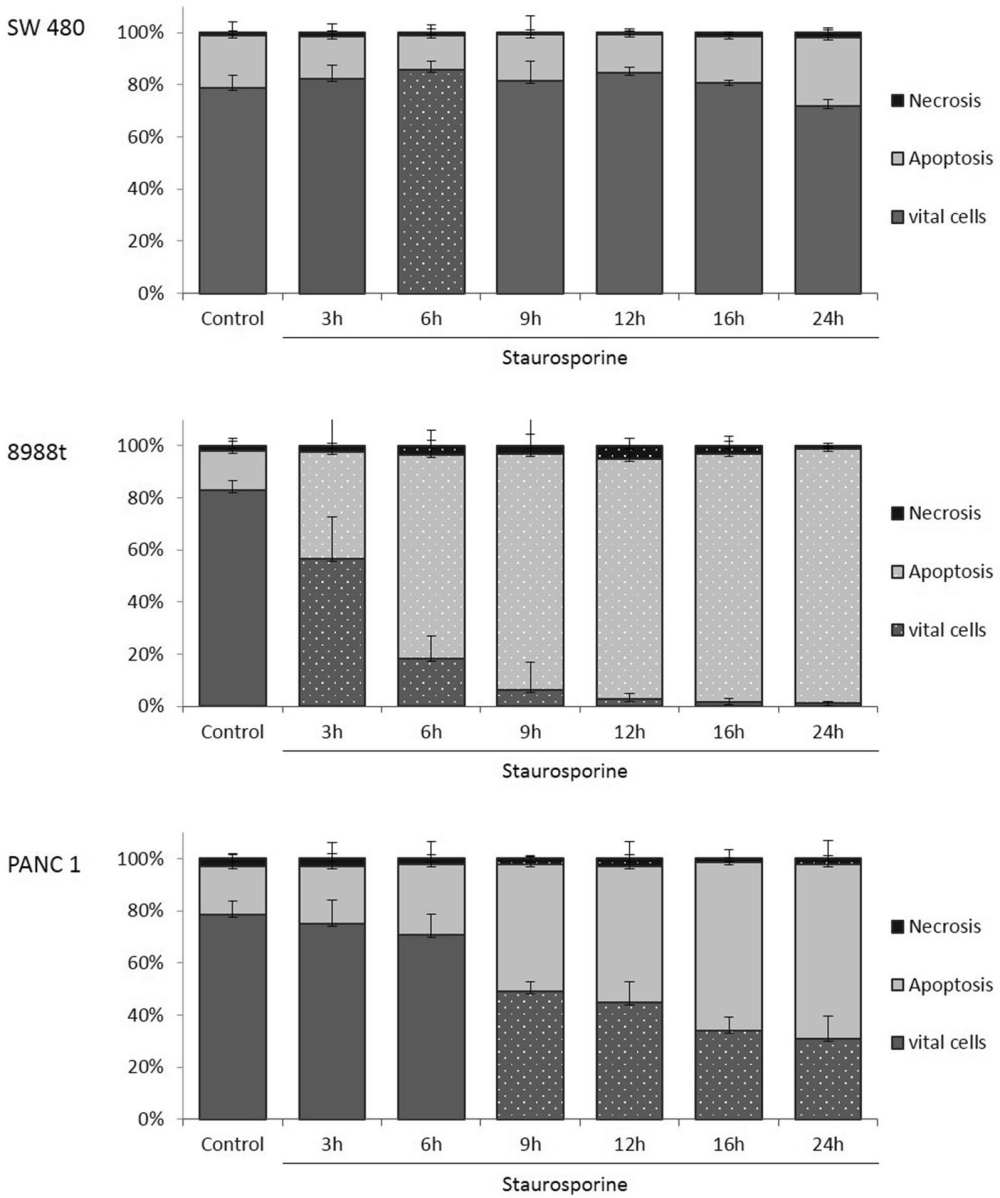
The effects of staurosporine on apoptosis in in vitro SW 480 colorectal carcinoma (**a**) and PaTu 8988t (**b**) and Panc-1 (**c**) pancreatic carcinoma cell lines after time-dependent incubation. For apoptosis analysis, cancer cells were stained with annexin V. (*) indicates statistical significance at *p* < 0.05 compared to untreated control

The annexin V staining apoptosis assay was used to determine whether incubation with staurosporine induced apoptosis or necrosis. Incubation with staurosporine for 6 h (Fig. 2a) increased the vital cell fraction phase of colorectal carcinoma cells SW 480 to 84.75% ± 3.57% compared to the untreated samples. No other significant changes in apoptosis rate or cell death behavior were observed during any of the other time frames.

In contrast to the untreated control samples in the pancreatic cancer cell line PaTu 8988t, incubation with staurosporine between 3 h and 24 h significantly increased the rate of apoptosis (Fig. 2b) and significantly reduced the number of vital cells. The necrosis rate was increased after 6 h, 12 h, and 16 h incubation. In Panc-1, stimulation with staurosporine (Fig. 2c) significantly increased apoptosis and significantly reduced the number of vital cells after 9 h, 12 h, 16 h, and 24 h.

### Endogenic expression of Bcl2, Bad, BAX, caspase-8, and caspase-9 in pancreatic and colorectal carcinoma cells

**Fig. 3:**
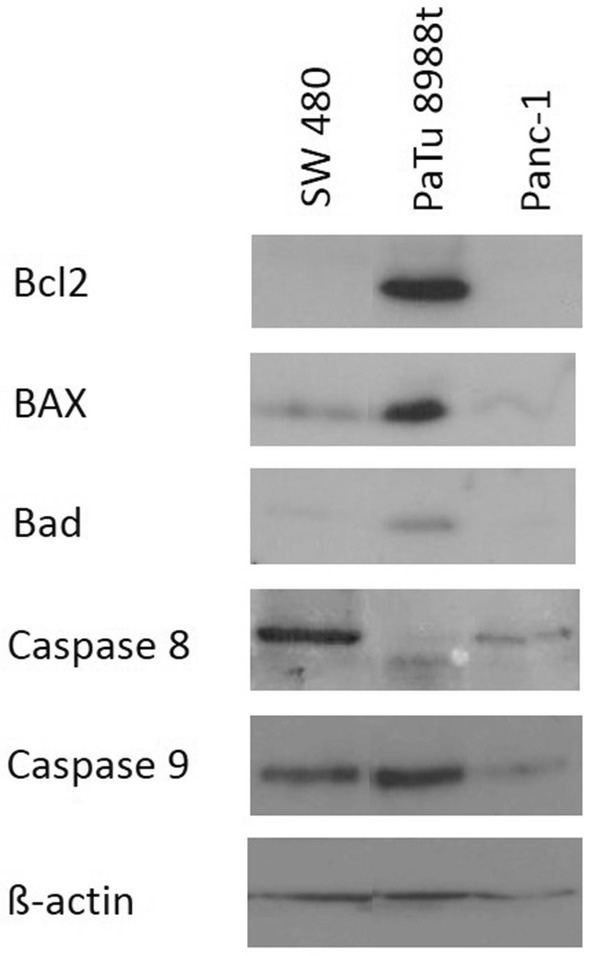
Immunblotting and proof of endogenic expression of Bcl2, BAX, Bad, caspase-8, caspase-9, and ß-actin in colorectal cancer cells (SW 480) and pancreatic cancer cells (PaTu 8988t and Panc-1)

The first aim was to obtain evidence for the actual expression of Bcl2, Bad, BAX, caspase-8, and caspase-9 in pancreatic and colorectal carcinoma cells (Fig. 3). The pancreatic cancer cell line PaTu 8988t (column 2) showed strong expression of each of the proteins investigated, whereas the cell lines SW 480 and Panc-1 showed only expression of BAX, caspase-8, and caspase-9. The proteins Bcl2 and Bad could not be detected at all. The endogenous expression of ß-actin serving as loading control can be seen in the lower blot (column 6).

### Western blot analysis after time-dependent incubation with 1 µM staurosporine and endogenic expression of Bcl2, BAX, Bad, caspase-8, and caspase-9 in pancreatic and colorectal carcinoma cells

**Fig. 4:**
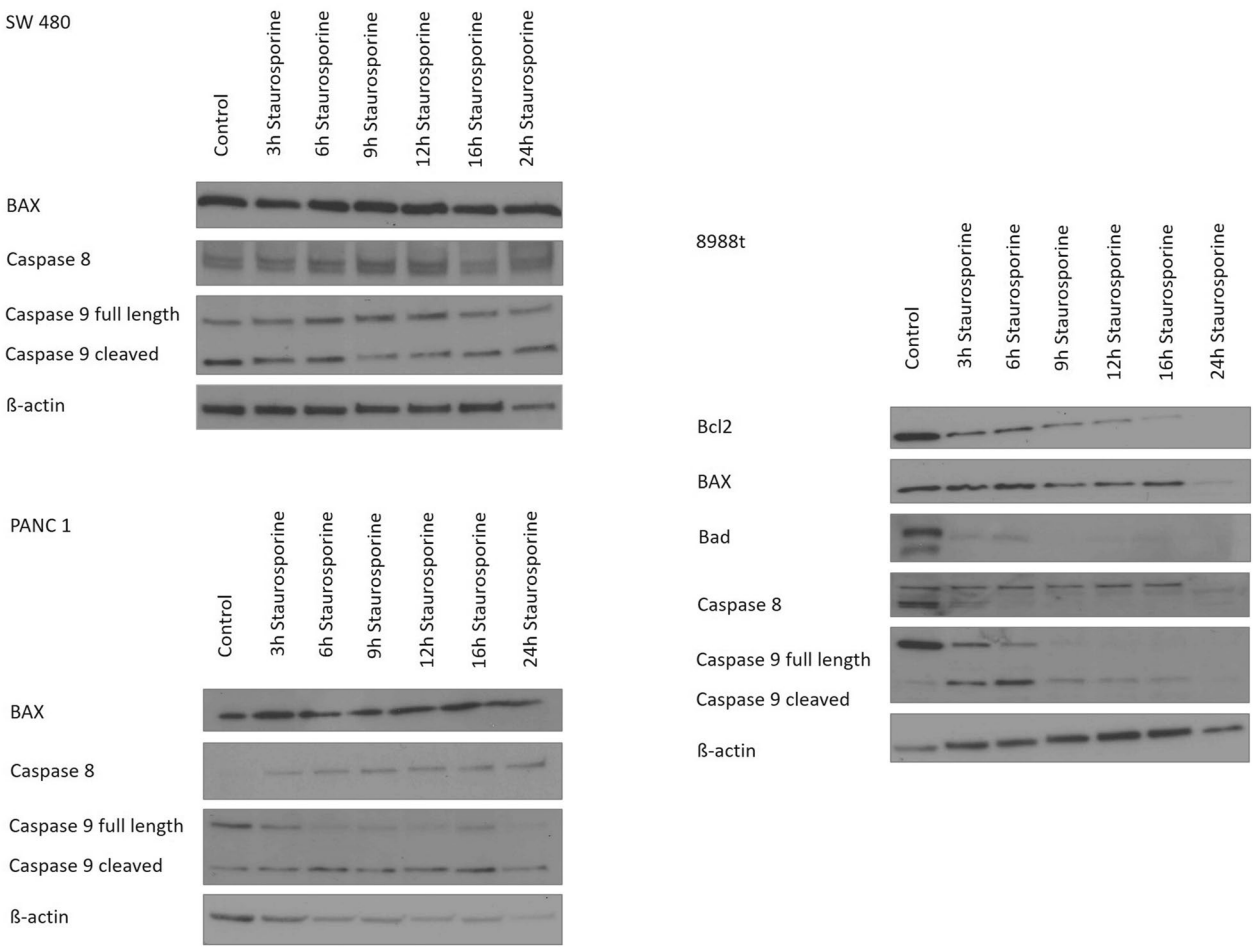
Time-dependent immunoblotting and proof of endogenic expression of Bcl2, BAX, Bad, caspase-8, caspase-9, and ß-actin in colorectal carcinoma cells (SW 480) and pancreatic cancer cells (PaTu 8988t and Panc-1) after stimulation with staurosporine

The colorectal cancer cell line SW 480 did not show any time-dependent changes in the expression of the proteins BAX, caspase-8, and caspase-9 (Fig. 4a). The pancreatic cancer cell line PaTu 8988t (Fig. 4b) showed a time-dependent decrease in the signal strength of Bcl2 after incubation with staurosporine up to the complete absence of proteins after 24 h of incubation (column 1). In contrast, expression of BAX and caspase-8 was not influenced by staurosporine; here, only the band intensity was decreased after 24 h of incubation (column 2 and 4). Expression of Bad was considerably decreased after 3 h and 6 h of incubation in the reagent in contrast to untreated cells only incubated in the medium. After 9 h of incubation, protein was no longer detectable (column 3).

Use of the antibody caspase-9 enabled detection of full length caspase-9 as well as cleaved caspase-9. The control samples, which had only been incubated in the medium, showed considerable signal strength for full length caspase-9 that was markedly decreased after 3 h and 6 h of incubation with staurosporine. At the later time points (9 h to 24 h of incubation), protein was no longer detectable. In contrast, expression of cleaved caspase-9 first increased after incubation with staurosporine, showing the maximum signal strength after 6 h of incubation, but steadily decreased over the later time points (column 5). Column 6 depicts the endogenous expression of ß-actin that served as loading control.

In the pancreatic cancer cell line Panc-1 (Fig. 4c), BAX expression was not influenced by incubation with staurosporine (column 1), whereas the signal strength of caspase-8 steadily increased in a time-dependent manner (column 2). Expression of full length caspase-9 decreased, but expression of cleaved caspase-9 increased after stimulation with the reagent. After 24 h of incubation, the signal strength of both bands was decreased. Column 4 depicts the endogenous expression of ß-actin that served as loading control.

## Discussion

Apoptosis is a key regulator of physiological growth control and tissue homeostasis. One of the most important findings in cancer research of the past few years is that apoptosis has a high impact on the development of tumors as well as on their response to chemotherapy [22, 23]. Bcl2 proteins residing on the mitochondrial membrane serve a regulatory function in the intrinsic apoptotic signaling pathway. They strictly control this pathway by inducing mitochondrial outer membrane permeabilization (MOMP). All proteins of the family show similar structural domains, called “Bcl2 homology (BH) domains” (named BH1, BH2, BH3, and BH4). They can be divided into three subgroups: the prosurvival members (Bcl2, Bcl-xl, Bcl-w, MCL-1, and A1), the pro-apoptotic members, which include the MOMP effectors (mainly BAX and BAK), and the BH3-only proteins, so called because they have only the BH3 domain (Bad, BIM, BID, PUMA, NOXA) [24].

Pro-apoptotic factors such as BAX, BAK, and Bad and the anti-apoptotic factors Bcl2 and Bcl-xl are delicately balanced, and this balance is often lacking in tumor cells [25]. For instance, dysfunction of BAX may further the tumor genesis of cells; thus, many chemotherapies try to indirectly intervene in this process [26]. Derivatives such as oblimersen sodium, AT-101, ABT-263, and GX15-070 are currently under clinical investigation [27, 28]. Furthermore, kinase inhibitors are also constantly used in cancer treatment [29, 30].

A highly potent inductor of apoptosis is staurosporine, an alkaloid that inhibits many different kinases [17]. In 2001, Stepczynska et al. [18] showed that staurosporine—as a broadband kinase inhibitor—induced apoptosis in Jurkat cells resistant to chemotherapeutic agents. Chae et al. [31] reported on the apoptosis-inducing effect of staurosporine in osteoblasts and Xue et al. [32] on this effect in breast carcinoma cells. The inhibiting effect of staurosporine on cell adhesion, mobilization, and invasion could also be shown in lung carcinoma cells [33]. To different degrees, staurosporine also seems to affect the induction of apoptosis in pancreatic and colorectal carcinoma cells. However, the signaling pathway by which staurosporine induces apoptosis has yet remained unclear.

The current study shows that—in the pancreatic and colorectal cancer cell lines Panc-1, PaTu 8988t, and SW 480—staurosporine does not influence BAX, but influences the anti-apoptotic factor Bcl2. The Bcl2 protein is often overexpressed in several types of cancer, for instance in breast, lung, and ovarian cancer or in malignant melanoma. Therefore, evidence of Bcl2 in cells is often associated with unfavorable outcome [34].

Bcl2 is expressed in the pancreatic cancer cell line PaTu 8988t. If Bcl2 is produced, apoptosis may be induced by suppressive medication. For this reason, the effect of BH3-mimetica has been repeatedly investigated in clinical trials, because these drugs induce apoptosis by binding and inhibiting anti-apoptotic members of the Bcl2 protein family [35, 36].

However, Bcl2 expression decreased after the administration of staurosporine in PaTu 8988t carcinoma cells. But why does staurosporine, as a kinase inhibitor, cause activation of the intrinsic apoptotic pathway? A possible explanatory approach could be that the imbalance between BAX and Bcl2—thus between pro-apoptotic and anti-apoptotic factors—probably induces apoptosis. This hypothesis is supported by the activation of full length caspase-9, shifting the balance to cleaved caspase-9. Cell apoptosis is eventually induced by activation of the intrinsic signaling pathway via caspase-9.

Bad—a further important protein in the apoptosis process—stands for Bcl2 antagonist of cell death. This protein may develop pro-apoptotic effects due to the heterodimerization with anti-apoptotic factors, for instance Bcl2, by binding to and thus blocking Bcl2 [37]. After stimulation with staurosporine, expression of Bad cannot any longer be detected by Western blot analysis. A possible explanation may be heterodimerization of Bad and Bcl2 that further shifts the balance between pro-apoptotic and anti-apoptotic factors in favor of pro-apoptotic factors. But degradation pathways at the level of transcription, translation level, or protein modification/clearance would also be conceivable.

Yuste et al. investigated whether overexpression of Bcl2 can rescue cells from staurosporine-induced apoptosis. They found that overexpression of Bcl2 increased the resistance of cells to staurosporine up to 1 µM. At higher doses, cytochrome c release from mitochondria occurred, caspases were activated, and cells died by apoptosis.

They also examined whether caspase inhibitors could rescue the cells from apoptosis induced by staurosporine. For this question they used the noncompetitive inhibitor of caspases z-VAD.fmk. The addition of z-VAD.fmk delayed the staurosporine-induced cell death [38].

The Bcl2 protein family plays a key role in the process of the intrinsic apoptotic signaling pathway, because dysfunctional Bcl2 protein may result in the development of both tumor cells and resistance to chemotherapies [39]. As a kinase inhibitor, staurosporine seems to be able to particularly influence the process of the intrinsic apoptotic signaling pathway.

In summary, this study showed that staurosporine induces apoptosis in pancreatic carcinoma cells via the intrinsic signaling pathway. Staurosporine is therefore a suitable positive control for in vitro apoptosis tests for the pancreatic carcinoma cell lines PaTu 8988t and Panc-1. In the colorectal cancer cell line SW 480, stimulation with staurosporine did not induce apoptosis.

## Conclusion

Modern therapeutic strategies for tumor diseases target the efficient modulation of specific signaling and transcription pathways (for instance, VEGF antibodies [40], tyrosine kinase inhibitors in the treatment of chronic lymphatic leukemia [41], or EGFR antibodies in the therapeutic management of advanced colorectal carcinoma [42]). In this respect, the therapeutic potential of protein kinase inhibitors has been repeatedly discussed. Pharmaceutical companies increasingly focus on the development of new chemotherapies [43]. The approval of chemotherapies (for instance, imatinib for treating chronic myeloid leukemia, trastuzumab for treating breast cancer, or gefitinib and cetuximab for treating lung and colorectal cancer) has opened up new possibilities of treating different types of cancer [44]. A large number of kinase inhibitors are currently undergoing clinical development in both clinical and preclinical trials to analyze the potential of these substances for medical treatment [45]. However, their side effects and toxicity should be closely monitored because kinase inhibitors may also modulate important signaling cascades in healthy cells.

Ultimately, the basis and prerequisite of such new therapeutic approaches is a comprehensive knowledge of the respective carcinogenesis. The only types of cancer that may benefit from therapeutic protein kinase inhibitors are diseases marked by upregulation of the specific signaling pathways and thus by disturbed natural balance between mitosis and apoptosis. Furthermore, a combination therapy of different target-specific therapeutics will probably be required to avoid an increase in the proliferation rate or a decrease in the apoptosis rate of cells and thus the development of uncontrolled cellular growth [44].

The present work provides insight into the complexity of the Bcl2 family and the apoptotic pathways. Many further trials will be required to identify the underlying molecular mechanisms. Identifying and characterizing cellular receptors and their signal-transduction cascades will eventually help establish new therapeutic approaches in the treatment of pancreatic carcinoma, one of the most aggressive types of all cancers.

## Abbreviations

Bad: Bcl2 antagonist of cell death
BAX: Bcl2-associated X
Bcl2: B cell lymphoma 2
BH3: Bcl2 homology domain 3
EGFR: epidermal growth factor receptor
MOMP: mitochondrial outer membrane permeabilization
VEGF: vascular epithelial growth factor
